# The Efficacy of Contrast-Enhanced Endoscopic Ultrasound for Differentiating Mural Nodules from Mucus Clots in Branch Duct IPMN

**DOI:** 10.3390/diagnostics16101497

**Published:** 2026-05-14

**Authors:** Naoki Mita, Takuji Iwashita, Yuki Utakata, Takuya Koizumi, Yosuke Ohashi, Shota Iwata, Hironao Ichikawa, Kensaku Yoshida, Akinori Maruta, Shinya Uemura, Katsuhisa Toda, Nami Asano, Masaki Katayama, Tatsuhiko Miyazaki, Masahito Shimizu

**Affiliations:** 1 Department of Gastroenterology, Gifu Prefectural General Medical Center, Gifu 500-8717, Japan; mitanao8@yahoo.co.jp (N.M.); ichi.hiro.m.0814@gmail.com (H.I.); kensakuyoshidaky@gmail.com (K.Y.); 2 First Department of Internal Medicine, Gifu University Hospital, Gifu 501-1194, Japan; takuya.koizumi0305@gmail.com (T.K.); yosuke-ohashi14@hotmail.com (Y.O.); iwthalop120@yahoo.co.jp (S.I.); mrak5844@yahoo.co.jp (A.M.); ueshin550621@gmail.com (S.U.); shimizu.masahito.j1@f.gifu-u.ac.jp (M.S.); 3 Department of Gastroenterology, Shiga University of Medical Science, Otsu 520-2192, Japan; 4 Department of Gastroenterology, Chuno Kosei Hospital, Seki 501-3802, Japan; y217.bs@gmail.com (Y.U.); snsk_toda@yahoo.co.jp (K.T.); 5 Department of Pathology, Chuno Kosei Hospital, Seki 501-3802, Japan; 6 Department of Pathology, Gifu Prefectural General Medical Center, Gifu 500-8717, Japan; 7 Department of Pathology, Gifu University Hospital, Gifu 501-1194, Japan

**Keywords:** IPMN, CE-EUS, mural nodule

## Abstract

**Background/Objectives**: The presence of a mural nodule (MN) is one of the findings indicating malignant transformation of an intraductal papillary mucinous neoplasm (IPMN). It is difficult to distinguish true MNs from mucus clots (MCs) by endoscopic ultrasound (EUS) alone. This study aimed to evaluate the efficacy of contrast-enhanced (CE)-EUS for differentiating true MNs from MCs and carcinoma from adenoma. **Methods**: A total of 104 patients who were diagnosed as having branch duct-type IPMNs with MN-like structures by EUS and underwent CE-EUS between January 2016 and August 2022 were included. MN-like structures without perfusion on CE-EUS were defined as MCs and those with perfusion were defined as true MNs. This was a retrospective study with limited pathological confirmation, and diagnoses in non-surgical cases were based on imaging and follow-up. **Results**: CE-EUS showed MN-like structures with perfusion in 35 patients and without perfusion in 69 patients. Surgical resection was eventually performed in a total of 28 patients and the diagnostic sensitivity, specificity and accuracy of MNs among them were 100%, 66.7% and 96.4% in CE-EUS; 48%, 66.7% and 50% in CE-CT; and 61.9%, 33.3% and 58.3% in MRCP, respectively. Possible risk factors indicating malignancy were statistically evaluated and presence of an MN was the only significant factor. Among the 35 true MNs, the height of an MN in carcinoma was significantly higher than that of an adenoma. The ROC analysis for detecting carcinoma in true MNs showed an area under the curve of 0.92 with the optimal cut-off value of 7 mm. When this cut-off value was used for diagnosing carcinoma, the sensitivity, specificity, and accuracy were 94.1%, 83.3% and 88.6%, respectively. **Conclusions**: CE-EUS may be useful for differentiating true MNs from MCs, although diagnostic performance should be interpreted cautiously because most non-surgical cases lacked pathological confirmation.

## 1. Introduction

Intraductal papillary mucinous neoplasms (IPMNs) are cystic lesions of the pancreas that are detected with increasing frequency due to recent advances in diagnostic imaging [1]. IPMNs are known to be potentially malignant tumors, with atypia ranging from low-grade dysplasia to high-grade dysplasia or invasive carcinoma. Surgical resection should be considered for high-grade dysplasia, ideally before developing to invasive carcinoma, because its prognosis is poor once an IPMN develops to invasive carcinoma [2,3]. However, surgical treatment such as pancreaticoduodenectomy is highly invasive. Therefore, accurate preoperative diagnosis of an IPMN is important to determine appropriate management and avoid overtreatment.

The presence of mural nodules (MNs) is known as one of the findings indicating the malignant transformation of IPMNs [4]. Endoscopic ultrasound (EUS) is widely used to evaluate IPMNs as a reliable diagnostic tool because of its higher spatial resolution, and the detection rate for MNs using EUS is reported to be superior to that of CT and MRI [5,6]. However, it is occasionally difficult to distinguish true MNs from mucus clots (MCs) by EUS, because the appearances of MNs and MCs are similar on EUS. Contrast-enhanced EUS (CE-EUS) allowing visualization of microvascular blood flow by using ultrasonographic contrast agents has been developed and its efficacy in improving the qualitative diagnostic performance in pancreatic lesions has been reported [7,8,9,10]. CE-EUS has the possibility to differentiate true MNs from MCs accurately. Thus, this study was conducted to evaluate the efficacy of CE-EUS for differentiating true MNs from MCs and carcinoma from adenoma in branch duct-type IPMNs (BD-IPMNs).

## 2. Materials and Methods

### 2.1. Study Design and Patient Selection

This was a retrospective cohort study conducted between January 2016 and August 2022 at three tertiary care centers (Gifu University Hospital, Gifu Prefectural General Medical Center, Chuno Kosei Hospital). A database analysis, including all EUS procedures, was performed to identify the patients who met the following criteria: (1) patients who underwent EUS and CE-EUS for BD-IPMNs with MN-like structures; (2) patients who underwent surgical resection or, in non-surgical cases, patients whose follow-up imaging studies after more than 6 months from CE-EUS were available. However, patients with surgically altered upper gastrointestinal anatomy except for Billroth I were excluded. This study was conducted in accordance with the human and ethical principles of research set forth by the Helsinki guidelines. The study protocol was approved by the Institutional Review Board at each institution.

### 2.2. Equipment and Endoscopic Procedure

All EUS procedures were performed under moderate sedation using Midazolam and Pentazocine and were performed with echoendoscopes (GF-UCT260 or GF-UE260; Olympus, Tokyo, Japan or EG-580UT; Fujifilm, Tokyo, Japan) connected with an ultrasound system (ARIETTA850, ProSound F75, ProSound α10 or SU-1; Fujifilm). Microbubbles composed of perfluorobutane with a median diameter of 2–3 μm (Sonazoid^®^: Daiichi-Sankyo, Tokyo, Japan; GE Healthcare, Milwaukee, WI, USA) were used as the contrast agent. When the MN-like structure was detected during observation of an IPMN by B-mode EUS, the EUS mode was changed to the extended pure harmonic detection mode for CE-EUS. The ultrasound contrast agent was suspended in 2 mL of water and intravenously injected at 0.015 mL/kg body weight. Any perfusion on the nodule was evaluated after administration of the contrast agent.

### 2.3. Definitions

**Figure 1:**
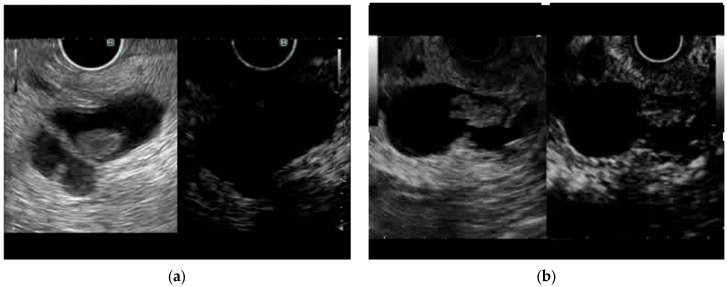
Definition of MC and true MN based on CE-EUS findings. (**a**) MN-like structure without perfusion on CE-EUS was defined as MC. (**b**) MN-like structure with perfusion on CE-EUS was defined as true MN.

An MN-like structure was defined as a solid component on the cyst wall that was recognized by EUS. MN-like structures with perfusion on CE-EUS were defined as true MNs and those without perfusion were defined as MCs (Figure 1). In patients who underwent surgical resection of IPMNs, low or intermediate dysplasia was defined as intraductal papillary mucinous adenoma (IPMA) and high-grade dysplasia or invasive carcinoma was defined as intraductal papillary mucinous carcinoma (IPMC). In patients who did not have surgical resection for an IPMN, the IPMN was defined as IPMA if there was no change on imaging findings of the IPMN or MN for more than 6 months. This definition represents a clinical diagnosis based on imaging and follow-up rather than pathological confirmation. The MN height was measured as the maximum vertical distance from the cyst wall to the apex of the MN. The adverse events were evaluated according to the American Society for Gastrointestinal Endoscopy workshop report [11].

## 3. Results

### 3.1. Patient and Lesion Characteristics

Between January 2016 and August 2022, 104 patients [52 male; median age, 71 years (range 44–85)] were enrolled in this study. The basic characteristics of the patients are shown in Table 1. The morphology of the IPMN was single cysts in 49 patients and multiple cysts in 55 patients, and unilocular cysts in 33 patients and multilocular cysts in 71 patients. The location of the cyst with an MN-like structure was the pancreatic head in 43 patients and the body and tail in 61 patients. The median diameters of cysts, main pancreatic ducts (MPDs) and MN-like structures were 20 mm (range 8–70 mm), 2.5 mm (range 0.9–30 mm) and 5.8 mm (range 2–49 mm), respectively. CE-EUS demonstrated MN-like structures with perfusion, which were defined as true MN in 35 patients, and without perfusion, which were defined as MCs in 69 patients. Among non-surgical cases, the median follow-up period was 28 months (range 6–75), with follow-up durations of 6–12 months in 12 patients, 12–24 months in 20 patients, and >24 months in 44 patients. No adverse events related to CE-EUS were recognized in this study.

### 3.2. Clinical Course and Diagnosis

**Figure 2:**
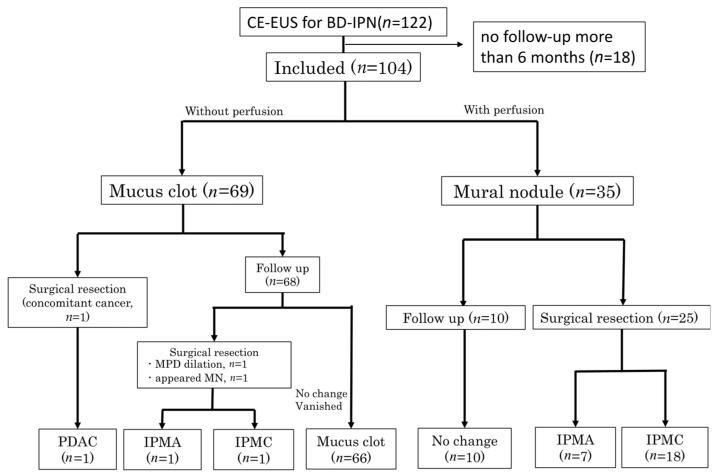
Patient flow and clinical course. A total of 104 patients with BD-IPMNs with MN-like structures were included. Patients were classified based on CE-EUS findings into true MN and MC groups. Surgical resection or imaging follow-up was performed.

Patient flow and details of the clinical course are shown in Figure 2. Among 35 patients diagnosed as having IPMN with MN, surgical resection was performed in 25 patients, whereas the remaining 10 patients underwent surveillance and showed no changes in imaging findings and were clinically diagnosed as having IPMA based on imaging and follow-up. As for 69 patients diagnosed as having IPMN with MC, surgical resection was performed due to concomitant pancreatic cancer in one patient. Among the remaining 68 patients, no changes were observed in IPMN by surveillance imaging studies conducted for more than 6 months in 66 patients, but surgical resection of the IPMN was performed because of dilated MPD (7.4 mm) in one patient and development of another MN with 13 mm height in one patient. MN-like structures that were diagnosed as MCs by CE-EUS did not become true MNs during follow-up as determined by CE-EUS or cross-sectional imaging. Surgical resection was eventually performed in a total of 28 patients and showed IPMC in 19 patients, IPMA in 8 patients and pancreatic ductal adenocarcinoma (PDAC) in 1 patient as the final pathological diagnosis. Among the 25 cases surgically resected for an MN, MN was confirmed by pathological examination of the resected specimen in 24 cases. In the remaining case, pathological examination showed a thickened cyst wall.

### 3.3. Diagnostic Performance of CE-EUS for Differentiation of MNs and MCs

Among the 28 surgically resected patients, the diagnostic sensitivity, specificity and accuracy of MNs were 100% (25/25; 95% CI 86.3–100%), 66.7% (2/3; 95% CI 9.4–99.2%) and 96.4% (27/28; 95% CI 81.7–99.9%) in CE-EUS; 48.0% (12/25; 95% CI 27.8–68.7%), 66.7% (2/3; 95% CI 9.4–99.2%) and 50.0% (14/28; 95% CI 30.6–69.4%) in CE-CT; and 61.9% (13/21; 95% CI 38.4–81.9%), 33.3% (1/3; 95% CI 0.8–90.6%) and 58.3% (14/24; 95% CI 36.6–77.9%) in MRCP (Table 2). Due to the retrospective nature of this study, a direct comparison of diagnostic performance among imaging modalities was not feasible, as CE-CT and MRCP were not performed in all patients. Diagnostic performance was evaluated only in surgically resected cases with pathological confirmation.

### 3.4. Risk Factor Indicating IPMC and MN Height Analysis

Possible risk factors indicating IPMC were statistically evaluated. In the univariate analysis, cyst size, presence of MNs and pancreatic head location were significant factors, whereas in the multivariate analysis, presence of an MN was the only significant factor (odds ratio: 24.8; 95% CI: 5.07–122.0) (Table 3).

**Figure 3:**
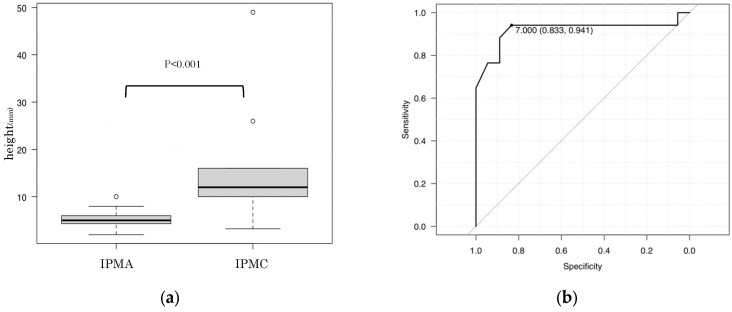
Statistical analysis of MN height: (**a**) Mann–Whitney U-test showed that the heights of MNs in carcinoma were significantly higher than those of adenoma. The box indicates the interquartile range, the horizontal line within the box indicates the median, and the whiskers indicate the minimum and maximum values excluding outliers. (**b**) The ROC analysis for detecting carcinoma in true MNs showed an area under the curve of 0.92 with an optimal cut-off value of 7 mm. The grey diagonal line indicates the reference line for random classification.

Regarding the MN height, among the 35 true MNs, the height of the MN in carcinoma was significantly higher than that of adenoma (carcinoma: 12 mm vs. adenoma: 5 mm; *p* < 0.001) (Figure 3). The ROC analysis for detecting carcinoma in true MNs showed an area under the curve of 0.92 with an optimal cut-off value of 7 mm. When this cut-off value was used for diagnosing carcinoma, the sensitivity, specificity, and accuracy were 94.1% (16/17; 95% CI 71.3–99.9%), 83.3% (15/18; 95% CI 58.6–96.4%) and 88.6% (31/35; 95% CI 73.3–96.8%), respectively. In addition, we performed the same analysis among the 28 surgically resected cases and ROC analysis for detecting carcinoma consistently showed an area under the curve of 0.87 with an optimal cut-off value of 7 mm. Using this cut-off value, the sensitivity, specificity and accuracy for diagnosing carcinoma were 84.2% (16/19; 95% CI 60.4–96.6%), 100% (9/9; 95% CI 66.4–100%) and 89.3% (25/28; 95% CI 71.8–97.7%), respectively.

## 4. Discussion

In this study, all MN-like structures that were diagnosed as MCs by CE-EUS did not become true MNs during follow-up period. This result suggested the possibility of differentiating MCs from true MNs by CE-EUS. Multivariate analysis showed that the presence of MNs was the only significant factor of carcinoma. In addition, the height of MNs on CE-EUS in carcinoma was significantly higher than that in adenoma among true MNs. The accuracy for malignancy was 88.6% when the cut-off value of 7 mm derived from ROC analysis was used. However, as most non-surgical cases were classified based on imaging follow-up rather than histopathological confirmation, the reported diagnostic performance of CE-EUS may have been overestimated due to potential verification bias and should therefore be interpreted with caution.

Generally, an MN is known as a factor indicating malignancy in an IPMN. Akita et al. retrospectively evaluated 38 patients diagnosed with a branch duct-type IPMN who underwent pancreatectomy and reported that an MN was a good predictor of malignancy and was identified as the only independent and significant marker of IPMC in multivariate analysis [12]. Furthermore, Ohno et al. recently reported that a systematic review of a total of 210 articles revealed a significant association between the presence of MNs ≥ 5 mm in diameter or solid components with contrast enhancement and the diagnosis of high-grade dysplasia or invasive carcinoma [13]. In our study, as previously reported, presence of MNs was the only significant factor in the multivariate analysis. The international consensus guidelines for IPMN defined enhancing MNs larger than 5 mm as high-risk stigmata (HRS) and recommended considering surgery if clinically appropriate [4]. Therefore, it is important to diagnose MNs accurately, and EUS is a useful modality for this purpose because of its high spatial resolution.

Among the 28 cases that underwent surgical resection in our present study, the diagnostic accuracy of MNs was 96.4% (27/28) in CE-EUS, 50% (14/28) in CE-CT and 58.3% (14/24) in MRCP, respectively. Because of the retrospective analysis, we could not directly compare the diagnostic capability between the examination modalities since we did not perform CT and MRI in all cases. However, this result suggests that CE-EUS might have higher accuracy than other cross-sectional imaging modalities. Similar to our study, the usefulness of EUS has been reported. A retrospective study by Harima et al. of 50 patients with IPMNs who underwent both CT and EUS revealed that the accuracy for diagnosing MNs was 92% using CT and 72% using EUS and it increased to 98% when EUS and CE-EUS were combined [14]. Yamashita et al. also reported that CE-EUS and conventional EUS showed significantly higher accuracy than CE-CT in the detection of MNs (92%, 83%, and 72%, respectively) [15]. These results suggest that EUS is a very useful modality for diagnosing the presence of MNs in BD-IPMN.

Despite the high detection rate of MNs with EUS, it is sometimes challenging to distinguish true MNs from MCs by B-mode EUS only. MCs can be depicted in a variety of forms and sometimes with findings similar to MNs on EUS. CE-EUS is very useful in differentiating MNs from MCs because it is able to visualize minute blood flow. In this study, CE-EUS was performed for 104 cases with MN-like structures on B-mode EUS, and all cases except for one false-positive case could be differentiated between MNs and MCs. In the only false positive case, a thickened cyst wall was identified in the pathological analysis, so we possibly misidentified the thickened cyst wall as an MN. Fujita et al. previously reported that they performed CE-EUS for 21 cases with MN-like structures detected by CT, MRI or fundamental EUS and 14 cases showed an avascular pattern. All 14 cases were diagnosed as having mucus lumps and were able to avoid surgical resection [6]. These results suggest that CE-EUS for MN-like structures is important for differentiating true MNs from MCs and for determining treatment strategy.

The size of the MN is also important when considering a management strategy for an IPMN. Although it is recommended to consider surgery for an IPMN with an MN larger than 5 mm in international guidelines, the optimal cut-off values of MN height for the prediction of malignant transformation vary among the reports [9,14,16,17]. Shimizu et al. reported that, as for MN heights measured by EUS, the diagnostic sensitivity and specificity for malignancy were 74.3% and 72.7%, respectively, when using the cut-off value of 7 mm [16]. This cut-off value was the same as that of our present study and diagnostic capability was higher in our study. The 7 mm threshold identified in our study may serve as an additional, exploratory parameter for risk stratification among true MNs, rather than replacing established guideline criteria. Therefore, this cut-off should be interpreted cautiously and in conjunction with existing guideline recommendations. The use of CE-EUS might have provided more accurate measurements of MN height and led to better diagnostic capability. Further studies to decide the optimal cut-off value of MN height to consider surgery are warranted.

From another point of view in evaluating the risk for malignancy in IPMN patients, various methods using cyst specimens have been reported in addition to morphological diagnosis in recent years. There are several reports including cystic fluid analyses such as cytology or examination of KRAS and GNAS mutation using EUS–fine needle aspiration [18,19,20] or utilization of artificial intelligence combined with EUS [21]. These studies are expected to further develop the prediction of malignant transformation of IPMNs in the future.

This study has several limitations. The primary limitation of this study is the lack of pathological confirmation in most cases. As the majority of non-surgical cases were classified based on imaging follow-up rather than histopathology, the diagnostic performance of CE-EUS may be overestimated due to verification bias. A retrospective study design with a limited sample size might cause biases in patient selection. Furthermore, the minimum follow-up period of 6 months may be insufficient to exclude slow-growing neoplasms.

## 5. Conclusions

CE-EUS showed potential utility in differentiating true MNs from MCs. MN presence was associated with malignant transformation, and an MN height cut-off of 7 mm may provide exploratory risk stratification. Prospective studies with pathological confirmation are warranted.

## Figures and Tables

**Table 1:** Baseline patient and lesion characteristics, management and pathological diagnosis.

	*n* = 104
Patient characteristics	
Gender (male/female), *n*/*n*	52/52
Age, median (range), year	71 (44–85)
Duration of follow up, median (range), month	28 (6–75)
Lesion characteristics	
Cyst size, median (range), mm	20 (8–70)
Cyst location (head/body and tail), *n*	43/61
Main pancreatic duct diameter, median (range), mm	2.5 (0.9–30)
Single or multiple cysts (single/multiple), *n*	49/55
Unilocular or multilocular cyst (uni/multi), *n*	33/71
CE-EUS findings	
MN-like structure size, median (range), mm	5.8 (2–49)
MN-like structure perfusion (yes/no), *n*	35/69
Management, *n*	
Follow up/Surgical resection	76/28
Pathological diagnosis, *n*	
IPMA/IPMC/Pancreatic ductal adenocarcinoma (PDAC)	8/19/1

**Table 2:** Diagnostic performance among the 28 surgically resected patients.

	CE-EUS % (*n*, 95% CI)	CE-CT % (*n,* 95% CI)	MRCP % (*n*, 95% CI)
Sensitivity	100% (25/25, 86.3–100)	48.0% (12/25, 27.8–68.7)	61.9% (13/21, 38.4–81.9)
Specificity	66.7% (2/3, 9.4–99.2)	66.7% (2/3, 9.4–99.2)	33.3% (1/3, 0.8–90.6)
Accuracy	96.4% (27/28, 81.7–99.9)	50.0% (14/28, 30.6–69.4)	58.3% (14/24, 36.6–77.9)

**Table 3:** Univariate and multivariate analyses of risk factors for malignancy in patients with IPMNs.

	Univariate Analysis		Multivariate Analysis	
	Odds Ratio ^1^ (95% CI)	*p* Value	Odds Ratio ^1^ (95% CI)	*p* Value
Gender, (male)	1.10 (0.42–3.08)	0.80		
Age, (year) *	1.01 (0.96–1.07)	0.70		
Largest cyst size, (mm) *	1.05 (1.01–1.09)	0.01	1.03 (0.98–1.08)	0.24
Presence of mural nodule, (yes)	31.6 (6.68–150.0)	<0.001	24.8 (5.07–122.0)	<0.001
MPD size, (mm) *	1.04 (0.94–1.16)	0.47		
Multilocular cyst, (yes)	0.76 (0.27–2.14)	0.60		
Multiple cyst, (yes)	0.59 (0.22–1.61)	0.30		
Located in pancreatic head, (yes)	2.99 (1.06–8.38)	0.04	1.48 (0.42–5.21)	0.54

^1^ The odds ratio of a continuous variable is the value when the continuous variable increases by one unit. * Continuous variables.

## Data Availability

The data supporting the reported results of this study are not publicly available due to privacy and ethical restrictions. The authors make data available upon request.

## Abbreviations

The following abbreviations are used in this manuscript:

IPMN	Intraductal papillary mucinous neoplasm
CE-EUS	Contrast-enhanced endoscopic ultrasound
MN	Mural nodule
MC	Mucus clot
BD-IPMN	Branch duct-type intraductal papillary mucinous neoplasm
IPMA	Intraductal papillary mucinous adenoma
IPMC	Intraductal papillary mucinous carcinoma
MPD	Main pancreatic duct
PDAC	Pancreatic ductal adenocarcinoma
95% CI	95% confidential interval
CE-CT	Contrast-enhanced computed tomography
MRCP	Magnetic resonance cholangiopancreatography
ROC	Receiver operating characteristic
